# A pipeline for monitoring water pollution: The example of heavy metals in Lombardy waters

**DOI:** 10.1016/j.heliyon.2022.e12435

**Published:** 2022-12-15

**Authors:** Marco Zanchi, Stefano Zapperi, Chiara Zanotti, Marco Rotiroti, Tullia Bonomi, Stefano Gomarasca, Stefano Bocchi, Caterina A.M. La Porta

**Affiliations:** a Department of Environmental Science and Policy, University of Milan, via Celoria 10, 20133 Milano, Italy; b Center for Complexity and Biosystems, University of Milan, Via Celoria 16, 20133 Milano, Italy; c Department of Physics, University of Milan, Via Celoria 16, 20133 Milano, Italy; d CNR - Consiglio Nazionale delle Ricerche, Istituto di Chimica della Materia Condensata e di Tecnologie per l'Energia, Via R. Cozzi 53, 20125 Milano, Italy; e Department of Earth and Environmental Sciences, University of Milano-Bicocca, Piazza della Scienza 1, 20126 Milan, Italy; f CNR - Consiglio Nazionale delle Ricerche, Istituto di Biofisica, via Celoria 10, 20133 Milano, Italy; g Innovation For Well-Being And Environment (CRC-I-WE), University of Milan, Via Celoria 10, 20133 Milano, Italy

**Keywords:** Pollutants, Heavy metals, Water, Geolocalization

## Abstract

Time-dependent geolocalized analysis of pollution data allows to better understand their dynamics over time and could suggest strategies to restore a good ecological status of contaminated area. This research analyzes concentrations of pollutants in surface waters and groundwater monitored by the Regional Environment Protection Agency of Lombardy from 2017 to 2020. Lombardy is one of the richest and populous region of Europe, providing an interesting example of the impact of environmental pollutants due to anthropogenic and industrial activities, not only for Italy but also for all Europe. Results show that groundwater displays more sites with heavy metals above the legal limit with respect to surface waters, including As, Ni, Cr and Zn. Furthermore, the spatio-temporal analysis of the data clearly shows that the introduction of more restrictive laws is a proper policy to improve the ecological status of the water.

## 1 Introduction

Urbanization and industrialization have increased waste production including heavy metals, leading to a significant negative impact on human health [1], [2], [3]. Heavy metals, which are compounds with high atomic weight or with density at least 5 times greater than that of water, are naturally present on the Earth. However, anthropogenic activities, such as industrial production or agricultural use of metal-containing compounds, increase environmental contamination and therefore human exposure [4], [5], [6], [7], [8], [9]. Acute or chronic poisonings by heavy metals are due to exposure through water, air, and food and their bioaccumulation can lead to various toxic effects on several body tissues and organs (for a more detailed review see [1]). In particular, the contamination of waters by the presence of pollutants harmful to human health is a critical issue worldwide [10], [11], [12]. Indeed, being non-degradable materials, their concentration continuously increases in water bodies becoming part of the food chain and the local environment [13] and intoxicating organisms far from the source of pollution [14], [15].

In order to stop and counter this phenomenon, it is important to have a clear view and comprehension of the spatio-temporal state of water pollution both on local and global scales. In the literature, there are studies which try to offer a picture of the water contamination state on both scales. Kumar et al. [11] and Zhou et al. [16] evaluated the content of heavy metals in surface waters, lakes and rivers from different countries around the world. Wu et al. [17] studied the trace metal pollution in surface water from Yangtze River in Nanjing Section, China. Jahan [18] assessed the quality of waters of Australian ports. Karaouzas et al. [19] offered a clear picture of heavy metal contamination status in Greek surface waters. A recent paper provided a study on the spatial distribution of heavy metals in Albania's soils [20]. On the other hand, the presence of undesired dissolved species in water can result from natural processes. It is widely known that geogenic reduced species currently threaten groundwater quality in several areas worldwide [21], [22]. The release of reduced species such as As, Fe and Mn in groundwater through natural processes is mainly due to reductive dissolution mechanisms [21] driven by organic matter degradation [23]. In areas affected by these kinds of processes, which is the case of the Lombardy region in Northern Italy, species such as Fe, As and Mn can naturally reach groundwater concentrations higher than threshold values for groundwater quality assessment and potability [24], which constitutes a major issue for water management and potabilization.

Lombardy is the richest, most populous, and productive region of Italy and the third most populous region in Europe, showing a GDP higher than those of many EU Member States. Due to this, it represents an important example for the impact of environmental pollutants due to anthropogenic and industrial activities for all Europe. In the framework of the EU Green Deal, the reduced use of chemicals in agriculture, transports and industry appears to be a goal of key importance. To develop the best policy and the needed corrections, it is important to have a clear picture of the state of pollution in the air, water and in the soil. However, the spatio-temporal state of water pollution by heavy metals in Lombardy has not been deeply tackled in the literature.

The main goal of the present paper is to develop an integrated strategy combining geolocalization and the quantification of heavy metals, alone or in combination, in samples of groundwater and surface water, using geolocalized data from the Regional Environment Protection Agency (ARPA) in Lombardy over the course of four years, from 2017 to 2020. The spatial and quantitative analysis provided by the present paper offers a clear and comprehensive picture of the state of the art of the presence and the dynamics of heavy metals in this important Italian region in the years between 2017 and 2020. Moreover, the temporal and spatial analysis allows us to identify the most polluted areas and their possible change over time. These important data, that frame with efficient synthesis and clarity a very complex phenomenon with many ecological, geological, social, and economic implications, represent an effective key to interpreting the heavy metals problem in the environment. Finally, this work offers valuable information to support new policies for improving the ecological status of waters and soils, as well as human health.

## 2 Materials and methods

### 2.1 Data

Data relating pollutants in Lombardy from 2017 to 2020 were provided by Regional Environment Protection Agency (ARPA). In particular, data concern raw water, without any potabilization treatment. ARPA-Lombardy monitors monthly 492 groundwater stations and 350 points placed along the main river network (temporary and small water bodies are excluded since they are often linked to the irrigation system). In the present research, only monitoring stations, which have provided measures in each year of interest (2017-2020), have been considered. The list of pollutants reported in the database is the one provided by the European and Italian Water Environmental Directives (Dir. 2000/60/EC, Dir. 2006/118/EC, Dir. 2008/105/EC, Dir. 2009/90/EC, M.D. 56/2009, M.D. 260/2010, Lgs. D. 152,/2006, Lgs. D. 30/2009, Lgs. D. 219/2010). Databases contain 545210 and 1033871 records for groundwater and surface waters, respectively. Among all recorded substances, only measures related to heavy metals have been selected. In particular, heavy metals species, which haven't been measured continuously in the years of interest (2017-2020), have been excluded from our analysis. For the analysis of groundwater As, Cr(VI), Fe, Mn, Ni and Zn have been considered. While for surface waters, As, Cr(VI), Ni and Cr have been taken into account [25].

### 2.2 Data analysis

Data were analyzed using a custom-made python 3.8 jupyter notebook. A statistical frequentist approach has been applied to analyze the heavy metals' concentration. In particular, the analysis focuses both on time and spatial domains. In the first case, concentrations averaged over the years of interest (2017-2020) have been computed. In the latter, the spatial variations of the measurements, averaging concentrations on each location of the monitoring stations, has been considered. The combination of these two approaches has allowed to describe the spatial-temporal variation of the water contamination in Lombardy. In addition, the co-occurrences of the measurements of each pair of heavy metal species have been computed, counting the number of different monitoring stations which measure them simultaneously.

### 2.3 Data visualization

Geolocalized data for shape files and municipalities borders were obtained from the geoportal of the Lombardy region and plotted using python (3.8) with the geopandas (0.6.3), matplotlib (3.5.0) and seaborn (0.11.2) packages. Boxplots and heatmaps were plotted using the seaborn package.

### 2.4 Study area

**Figure 1:**
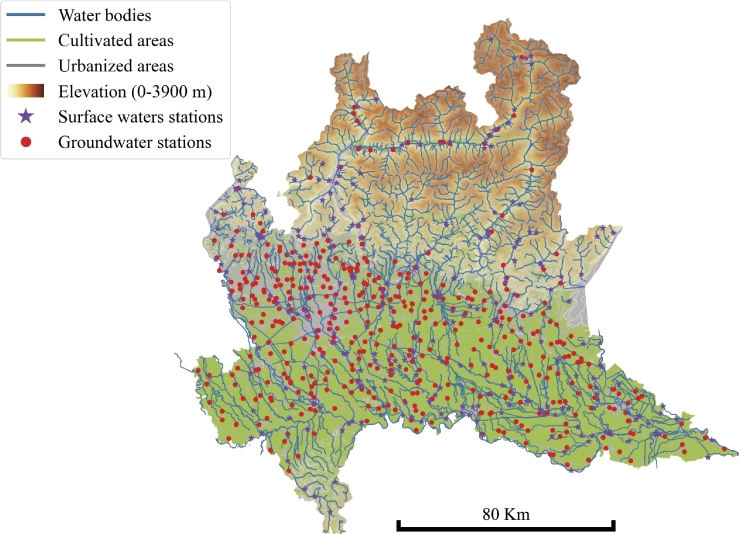
**Map of the study area of Lombardy region, Italy.** The figure describes the study area: Lombardy. The morphological and hydrological features are represented with the locations of groundwater and surface waters monitoring stations.

The present research focuses on Lombardy since it is one of the most developed regions in Italy and Europe. This area extends for ca. 29000 km^2^ and hosts around 10 million people highly concentrated around the big metropolitan area as Milan, Varese, Como, Bergamo, Brescia, Mantova and Lodi. Lombardy landscapes and culture have been highly influenced by intensive agriculture and industrial activities, leading to a strong urbanization and to the presence of pollutants in soils and waters. In Lombardy, there are three distinct natural zones: going from north to south, there is an Alpine area and an alluvial plain area which is further divided into high and low plains. The Alpine region hosts several unconfined local Alpine Valley Aquifers (AVA). The alluvial plain is mainly composed of Pleistocene sediments engraved by river valleys covered by Holocene fluvial sediments. The higher plain hosts a mono-layer structure mainly made of sand and gravel. In the southern area, the lower plain hosts a more stratified structure generated by the intercalation of silty-clayey into sandy layers [26]. Silt and clay layers in the lower plain are commonly accompanied by buried peats which degrades promoting reducing conditions. The alluvial plain aquifer system is composed of three superimposed aquifers: the Shallow Po Plain Aquifer (SPPA), the Intermediate Po Plain Aquifer (IPPA) and the Deep Po Plain Aquifer (DPPA) (Piano tutela delle acque, Lombardy, 2016). The SPPA is more than 100 m thick in the most northern area and becomes around 50 m thick in the higher plain, decreasing to 20–30 m in the lower plain. The shallow aquifers are unconfined in the higher plain and semiconfined/confined in the lower plain. The depth of the groundwater table is higher in the higher plain, with a thicker unsaturated zone, while it is very low in the lower plain. The IPPA is around 50–100 m thick in the higher plain, where it is unconfined/confined, and it gradually thickens, up to 600 m, in the lower plain, where it is unconfined. The DPPA is tapped by water wells only in the north-west area, where the overlying SPPA and IPPA have lower thickness. The climate of the study area is mainly humid subtropical (“Cfa” according to the Koppen and Geiger classification), with warm and wet summers and cold winters. However, the Lombardy region exhibits significant climate variations with respect to the Koppen model due to local variability in elevation, proximity to large water basins, and metropolitan areas. The annual average temperature is around 13.1 ^∘^C (55.5 ^∘^F), and the average annual rainfall is around 853 mm. The pluviometric regime, in particular, shows two minima in winter and summer and two maxima during spring and autumn seasons. Fig. 1 describes the study area.

## 3 Results and discussion

### 3.1 Geolocalization of heavy metals in surface water and groundwater in Lombardy from 2017 to 2020

“Big Data” analysis approaches over geolocalized data offer the possibility to identify part of lands particularly polluted and, therefore, to propose possible remedies. According to this, the quantity of metals in surface waters and groundwater recorded by ARPA in Lombardy in a period between 2017 and 2020 has been investigated. Analyzed metals represent the 3% of the total pollutants analyzed for surface waters and 2% for groundwater. The overall dataset includes a total of 18889 records for groundwater and 23385 records for surface waters for the entire period considered.

**Figure 2:**
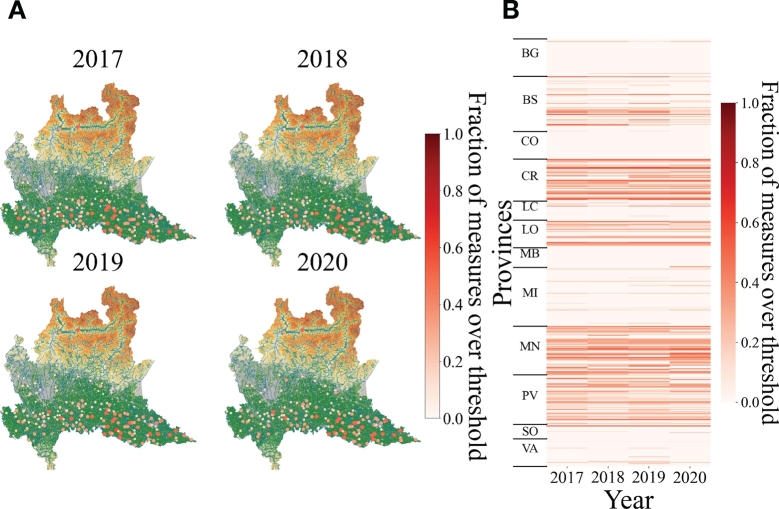
**Spatial distribution of heavy metals in groundwater of Lombardy.** A) Geospatial heatmap of the presence of heavy metals measured in groundwater of Lombardy for different years. Each point represents the fraction of measures which exceed the legal threshold concentration on a particular monitoring station. When a point is colored in dark red it means that the recording stations have measured more frequently concentrations of heavy metals over the legal threshold value. When a point is colored in light yellow it means that the recording stations have measured more frequently concentrations of heavy metals under the legal threshold value. If the fraction of measures which exceed the legal threshold concentration on a particular monitoring station is zero the point is not represented. B) Heatmap of the fraction of measurements of the concentration of heavy metals in groundwater of Lombardy over the legal threshold value divided for municipalities, provinces and years. Y-axis presents all the municipalities distributed by provinces while the x-axis represents the years.

Fig. 2A describes the geospatial heatmap of the presence of heavy metals measured in groundwater by ARPA monitoring stations for the different years. The size and the color of the points represents the fraction of measurements which overcome the legal threshold concentration. Fig. 2B displays the heatmap of the fraction of measurements of the concentration of heavy metals in groundwater of Lombardy over the legal threshold value divided for municipalities, provinces and years. Table 1 and Table 2 report the number of total measurements available for each of the studied species, the considered legal threshold values and the number of measurements exceeding those threshold values respectively for underground waters and surface waters. The data presented in Table 1 highlight that Ni and Zn contribute only marginally to the total pollution with respectively 11 and 17 samples exceeding the regulatory limit, while Cr, Fe, Mn and As are more significant.

**Table 1:** Groundwater information.

Heavy metals	Total measurements	Threshold [μg/L]	Measurements over threshold	Monitoring stations with measurements over threshold
As	3116	10	290	51
Cr(VI)	3094	5	72	20
Fe	3195	200	540	109
Mn	3196	50	918	158
Ni	3143	20	11	7
Zn	3145	3000	17	5

The table shows some information about the number of total measurements, the legal threshold concentration, the measurements over the legal threshold and the number of monitoring stations which measures concentration over the legal threshold from groundwater. The thresholds have been selected from D.Lgs. 152/2006.

**Table 2:** Surface waters information.

Heavy metals	Total measurements	Threshold [μg/L]	Measurements over threshold	Monitoring stations with measurements over threshold
As	5713	10	163	28
Cr(VI)	2994	5	45	6
Ni	7380	20	182	19
Cr	7298	50	6	2

The table shows some information about the number of total measurements, the legal threshold concentration, the measurements over the legal threshold and the number of monitoring stations which measures concentration over the legal threshold from surface waters. The thresholds have been selected from D.Lgs. 152/2006.

It is clear by Fig. 2 that the south of Lombardy (the Lower Plain) presents higher concentrations of heavy metals. The presence of heavy metals seems to remain stable from 2017 to 2020. However, in general the monitoring stations seem to measure quite rarely concentrations over thresholds, as pointed by smooth reds color in Fig. 2. Indeed, the average percentage of exceeding the legal threshold concentration over the monitoring locations and over the years is around 25% with a standard deviation of the mean of 14%. This can be linked to the increase of the taken measures carried on by ARPA. Monitoring activity is increasingly punctual and rigorous over time, in particular after the Legislative Decree 260/2010 approval, which entered into fully effective in the February 2011 (the L.D. 260/2010 updates the L. D.152/2006 “Environmental Regulations”, which in turn transposes the European Water Framework Directive -2000/60/EC). This Decree introduces new technical criteria for the classification of the water bodies ecological status and, in the case of groundwater and surface waters, criteria for the identification of a suitable monitoring network, as well as the ecological indicators and the limit values of the pollutants that can characterize groundwater, surface water, as well as transition water.

**Figure 3:**
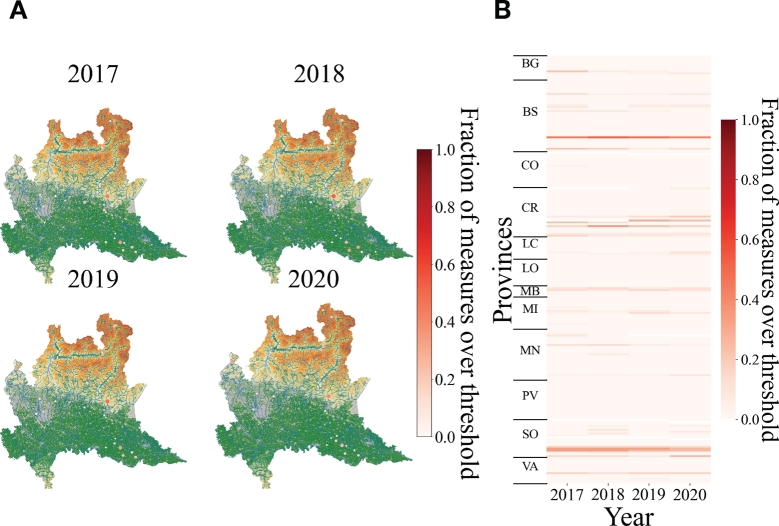
**Spatial distribution of heavy metals in superficial waters of Lombardy.** A) Geospatial heatmap of the presence of heavy metals measured in superficial waters of Lombardy for different years. Each point represents the fraction of measures which exceed the legal threshold concentration on a particular monitoring station. When a point is colored in dark red it means that the recording stations have measured more frequently concentrations of heavy metals over the legal threshold value. When a point is colored in light yellow it means that the recording stations have measured more frequently concentrations of heavy metals under the legal threshold value. If the fraction of measures which exceed the legal threshold concentration on a particular monitoring station is zero the point is not represented. B) Heatmap of the fraction of measurements of the concentration of heavy metals in superficial waters of Lombardy over the legal threshold value divided for municipalities, provinces and years. Y-axis presents all the municipalities distributed by provinces while the x-axis represents the years.

Fig. 3A-B shows that the concentration of heavy metals in surface waters does not exceed frequently the maximum permissible limits. Indeed, the average percentage of exceeding the legal threshold concentration over the monitoring locations and over the years is a about 14% with a standard deviation of the mean of 11%. Comparing with respect to groundwater, heavy metals appear to exceed the legal threshold value less frequently.

**Figure 4:**
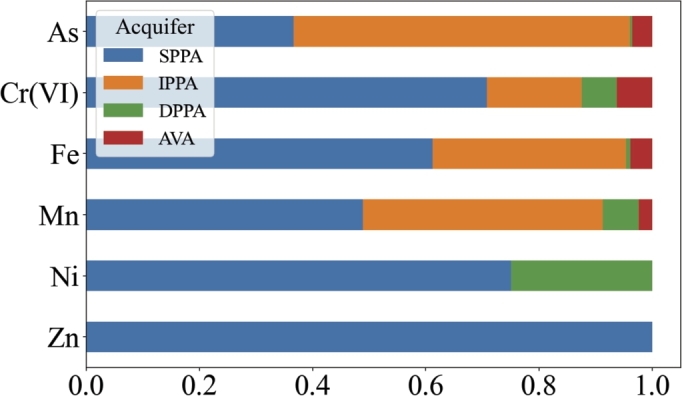
**Percentage distribution of metals in aquifers** The plot represents the distribution of the recordings for each metal in the different kind of aquifers. Only the samples exceeding the threshold have been taken into account. The abbreviations for the aquifers names are explained in the study area section.

Regarding groundwater, Fig. 4 shows how Zn, Ni and Cr(VI), mostly associated with anthropogenic processes, are detected in most cases in the shallow unconfined aquifer, which is more vulnerable to contamination related to land use. On the other hand, Fe, As and Mn which are mostly related to geogenic redox processes [23] are consistently present also in deeper aquifers. It is possible to notice that the majority of measurements are recorded in the SPPA and IPPA, respectively the 65% and the 25%.

### 3.2 Heavy metals present above the legal limit and co-localization in Lombardy between 2017-2020

The research focuses not only on which heavy metals were measured above the legal threshold concentration but also on which of these were detected together in the same location.

**Figure 5:**
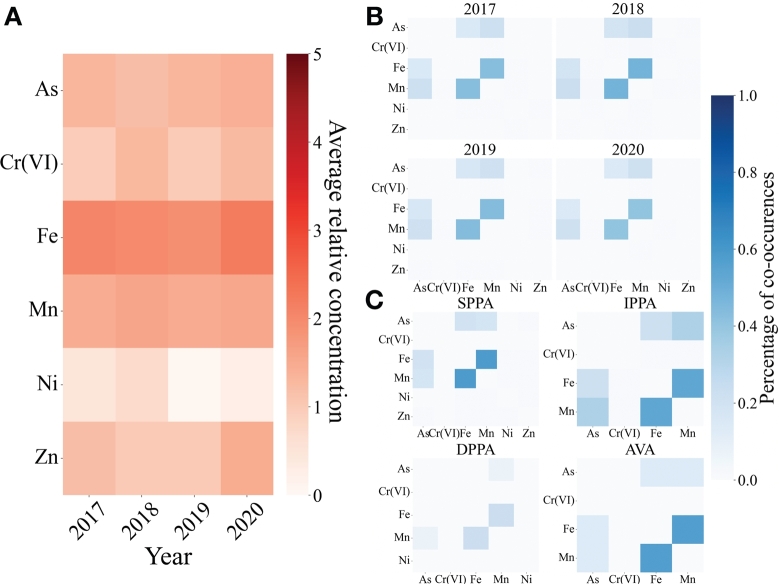
**Analysis of the heavy metals species in groundwater of Lombardy, considering only the samples exceeding the legal threshold value** A) Heatmap of the average relative concentration of the heavy metals species divided for years. Y-axis presents the metals species, while the x-axis represents the years. The plot shows that the metals which have higher concentrations over the legal threshold values are Fe and Mn. B) Co-occurrence matrices for the metals species detected in groundwater of Lombardy for different years. Each matrix shows the percentage of simultaneously measurements of two metals in a monitoring station for each year. The percentage has been computed dividing the number of the co-occurrences in a year by the total number of monitoring stations. C) Co-occurrence matrices for the metals species detected in groundwater of Lombardy for different aquifers. Each matrix shows the percentage of simultaneously measurements of two metals in a monitoring station for each aquifer. The percentage has been computed dividing the number of the co-occurrences in an aquifer by the total number of monitoring stations.

Fig. 5A displays the heatmap of the average relative concentration over legal threshold of the heavy metals species for the 2017-2020 period for groundwater, considering only the samples over the threshold limit presented in Table 1. The relative concentration over legal threshold is defined as the logarithmic ratio of the measured concentration and the legal threshold value. The plot shows that the considered heavy metals (As, Cr(VI), Fe, Mn, Ni and Zn) don't show a clear increase in the selected period. However, it is possible to notice that Ni concentration seems to decrease from 2019 and Zn shows a peak of concentration in 2020. In order to identify which substances were co-present above the legal limit in the same place, cross-correlation matrices of percentage of co-occurrences have been represented in Fig. 5B for different years and in Fig. 5C for different aquifers (the percentage of co-occurrences has been computed dividing the number of the co-occurrences in a year by the total number of monitoring stations). This analysis highlights the different processes governing the presence of the different metals in groundwater. Indeed, Ni, Zn and Cr which are mostly anthropogenic do not have a strong correlation with other metals (the percentages of co-occurrence don't exceed the 1%), even if considering only the shallow aquifer, which indicates different sources. In contrast, Fe, Mn and As are strongly correlated, which indicates that they are mostly related to a common source. Fe and Mn reach a maximum co-occurrence frequency of 48% in 2018 and of 58% in SPPA aquifers. As and Mn reach a maximum co-occurrence frequency of 24% in 2018 and of 33% in IPPA aquifers. Fe and As reach a maximum co-occurrence frequency of 19% in 2018 and of 22% in IPPA aquifers. Particularly, they are associated to the reducing conditions determined by the degradation of the natural organic matter of the peat layers and the mobilization of its byproducts [23].

**Figure 6:**
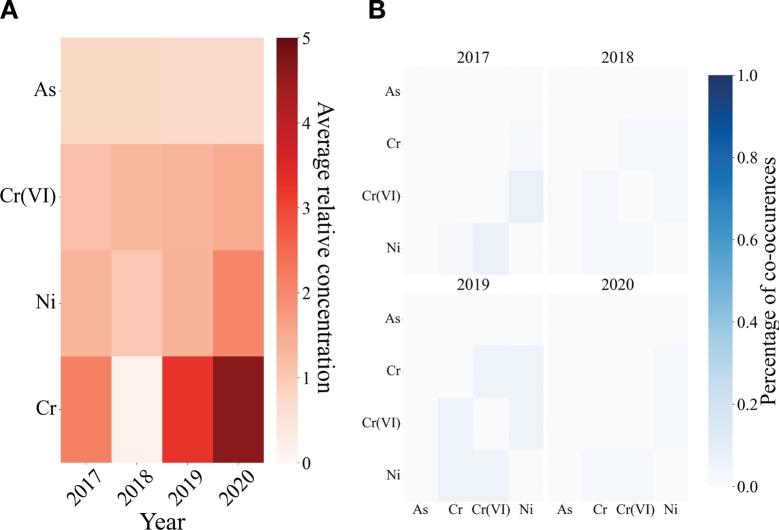
**Analysis of the heavy metals species in superficial waters of Lombardy, considering only the samples exceeding the legal threshold value.** A) Heatmap of the average relative concentration of the heavy metals species divided for years. The y-axis presents the metals species, while the x-axis represents the years. B) Co-occurrence matrices for the metals species detected in superficial waters of Lombardy for different years. Each matrix shows the percentage of simultaneously measurements of two metals in a monitoring station for each year. The percentage has been computed dividing the number of the co-occurrences in a year by the total number of monitoring stations.

The same analysis has also been carried out for surface waters, observing high concentrations of Ni and Cr (Fig. 6A). In particular, Cr seems to be measured in high concentrations in 2019 and 2020. Fig. 6B shows that there are not significant co-occurrences between the metals species for superficial waters. Indeed, the co-occurrence frequency values do not exceed the 10%.

**Figure 7:**
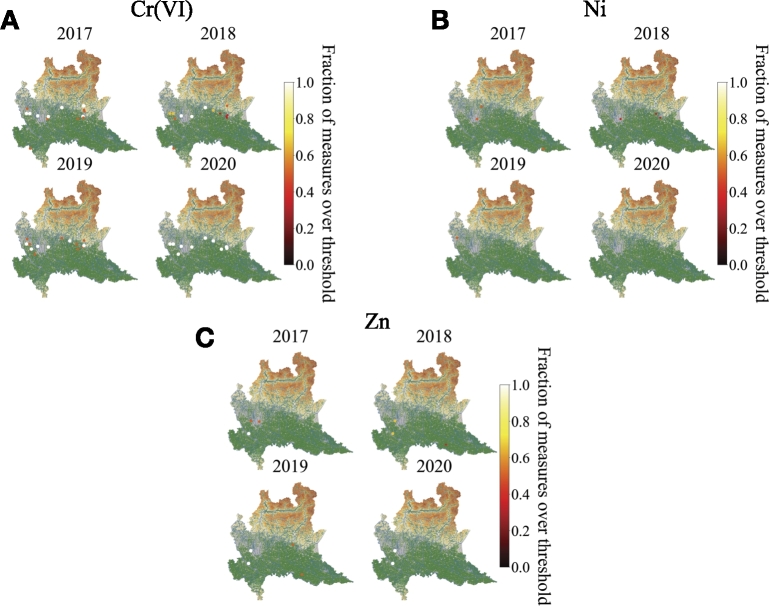
**Spatial distribution of Cr, Ni and Zn in groundwater of Lombardy.** Geospatial heatmap of the presence of A) Cr, B) Ni and C) Zn measured in groundwater of Lombardy for different years. Each point represents the fraction of measures which exceed the legal threshold concentration on a particular monitoring station. When a point is colored in white it means that the recording stations have measured more frequently concentrations of heavy metals over the legal threshold value. When a point is colored in dark red it means that the recording stations have measured more frequently concentrations of heavy metals under the legal threshold value. If the fraction of measures which cross the legal threshold concentration on a particular monitoring station is zero the point is not represented.

Furthermore, Fig. 7 represents the geolocalization map for Cr (Fig. 7A), Ni (Fig. 7B) and Zn (Fig. 7C) separately since those substances can have an anthropogenic origin. It is clear that Cr has been measured for all the years in a well establish area near the high industrialized area of Lombardy. A similar behaviour is shown by Zn which is measured every year in two locations. Instead, Ni does not show a regularity pattern during the years.

**Figure 8:**
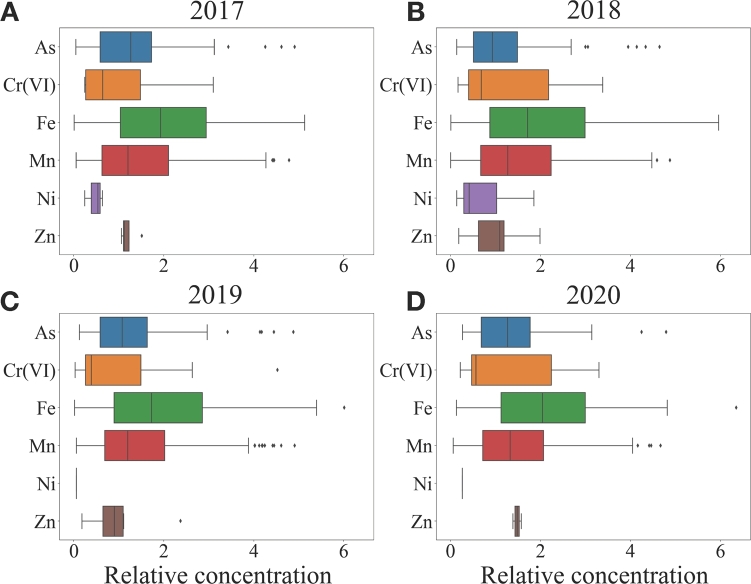
Boxplots of the relative concentration of the metals species in groundwater of Lombardy for different years considering only the samples exceeding the legal threshold concentration. Data refer to years A) 2017, B) 2018, C) 2019 and D) 2020. In the y-axis the different species of heavy metals are represented. While in the x-axis it is represented their relative concentration.

Finally, a barplot of the distribution relative concentration of the heavy metals detected in groundwater from 2017 to 2020 has been reported in Fig. 8A-D. It is possible to see that As, Fe and Mn reaches higher values of relative concentration respect to the other metals. This could be referred by their natural presence in groundwater in Lombardy, while the presence of high relative concentration for the other elements, in particular for Cr(VI), could be explained by anthropogenic activities. According to the previous results, the concentration distributions seem quite stationary over the years.

**Figure 9:**
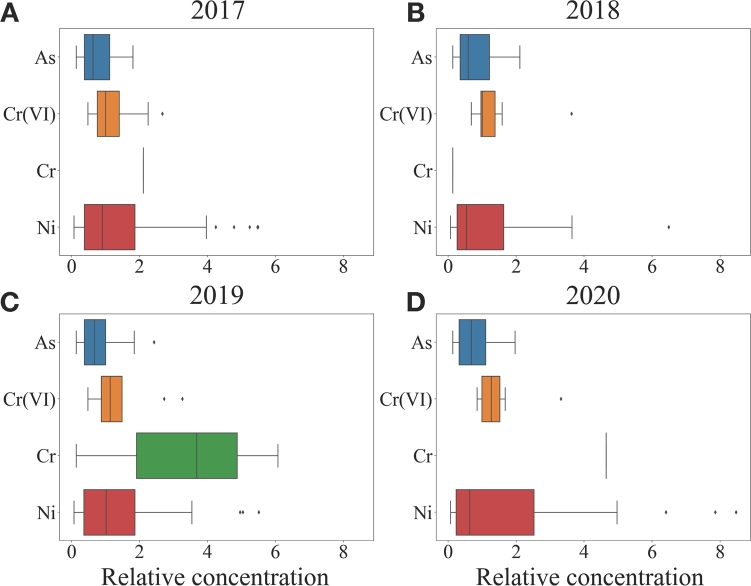
Boxplots of the relative concentration of the metals species in superficial waters of Lombardy for different years, considering only the samples exceeding the legal threshold concentration. Data refer to years A) 2017, B) 2018, C) 2019 and D) 2020. In the y-axis the different species of heavy metals are represented. While in the x-axis it is represented their relative concentration.

It is possible to observe a similar trend for surface waters as depicted in Fig. 9A-D. In particular, the relative concentration of Ni shows high and persistent values for all years.

### 3.3 Comparison of heavy metals concentration with their background natural value

**Figure 10:**
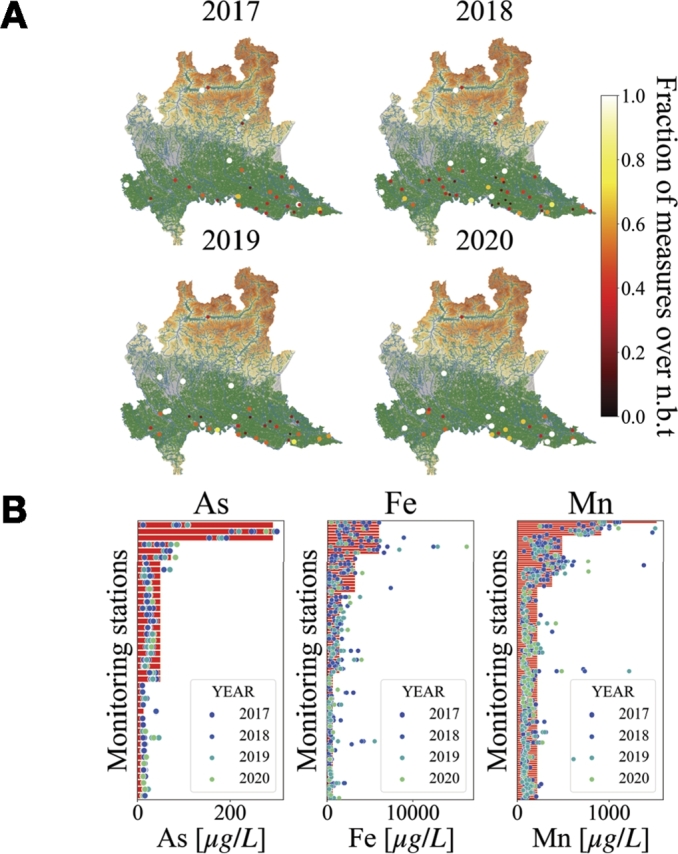
**Spatial distribution of As, Fe and Mn in groundwater of Lombardy respect to their natural background values.** A) Geospatial heatmap of the presence of heavy metals (As, Fe, Mn) measured in groundwater of Lombardy for different years. Each point represents the fraction of measures which cross the natural background threshold (abbreviated to n.b.t. in the colorbar label) on a particular monitoring station. When a point is colored in white it means that the recording stations have measured more frequently concentrations of heavy metals over the legal threshold. When a point is colored in dark red it means that the monitoring stations have measured more frequently concentrations of heavy metals under the natural background value. If the fraction of measures which cross the natural background threshold on a particular monitoring station is zero the point is not represented. B) The plot compares the distribution of measurements over the legal threshold (blue scatterplot) with the natural background value for each station (red bars). In the y-axis the monitoring stations are represented, while the x-axis measures the concentration. A plot is presented for each element (As, Fe, Mn).

Since As, Fe and Mn are naturally present in groundwater in Lombardy, the data collected by ARPA have been compared with their natural background values. The natural background values, evaluated in [24], vary from monitoring station to station and can be higher than the regulatory limit. The results are shown in Fig. 10. Fig. 10A represents the geospatial heatmap of the presence of As, Fe and Mn measured in groundwater by ARPA monitoring stations for the different years respect to their natural background concentrations. Fig. 10B describes the measured concentrations for all years (blue points) respect to the natural background value (red bars) for each monitoring station. The plot shows that most of the samples exceeding the regulatory limit can be associated to their natural background value, but still 12% of the samples exceed those natural background values (the percentage of points which surpass the red bar). Part of these cases can be associated to extreme values still related with the natural redox processes: since the natural background level calculation is based on the 95th percentile of each monitoring station and of each data population [24], it implies the that extreme values can still be found, higher than the natural background values. On the other hand, especially for those monitoring stations with a low natural background level, the samples exceeding the natural background level can be associated to an anthropogenic source. This is particularly evident in the higher plain, where groundwater is naturally in oxidizing conditions which should prevent the release of redox-sensitive species such as Fe, Mn and As.

### 3.4 Implications

Among the heavy metals present above the legal limit in groundwater As, Cr(VI), Ni and Zn appear to be the most dangerous when considering their possible impact on the environment and human health.

As is a natural component of the earth's crust but it is also employed industrially in materials processing during the production of glass, paper, textiles or as well as alloying agent. Other uses of As include diverse products such as pharmaceuticals, pesticides and feed additives. Due to its known toxicity, leading to serious health effects upon prolonged consumption [27], [28], [29], [30], As is a well studied groundwater contaminant. High concentrations of As in drinking water, leading to acute and chronic exposure, have been reported in numerous countries around the globe [28]. Contamination of groundwater by As has been already reported in Italy [31], [32]. In the present study, the highest level of As, with a value of 250 μg/L, has been found in Gabbioneta Binanuova close to Cremona in the eastern part of Lombardy in 2018. It has been reported that dissolved As concentrations are greater in groundwater compared to surface water. This happens since natural reducing processes, typical of Po plain groundwater, are related to longer residence times, while surface environments are mostly in oxidising conditions [33].

While Cr(III) is found in many vegetables and fruits and is an essential component of the human diet, the presence of Cr(VI) in the environment can be due both to natural causes, such as the erosion of natural Cr deposits, and anthropogenic causes related to industrial processes. These two forms of Cr can convert into each other and are therefore considered together when defining drinking water standards. The health effects of the ingestion of Cr(VI) are well known in the literature [34].

Ni concentrations in groundwater depend on a variety of factors such as soil use, sampling depth and pH. Increased Ni concentrations in groundwater and municipal tap water have been reported in many polluted areas [35]. Excessive amount of this metal was shown in the literature to have an adverse impact on the quality of the environment in terms of flora and fauna [36], and also an effect on human health [37]. Finally, Zn is an essential nutrient for body growth and development. Nevertheless, drinking water containing high levels of Zn was reported to be harmful for human health [38].

Finally, one important aspect that should be monitored over time is the co-presence of multiple pollutants in the same place. Indeed, they could generate a synergistic impact on the environment and therefore, when considering a “One health” approach, an impact to human health. This aspect was clearly shown in a recent paper [25] where the different effects of combination of pollutants present in the water have been tested on a biosensor, the alga *Chlamydomonas reinhardtii*.

## 4 Conclusion

This paper presents a detailed statistical analysis of the presence of heavy metals in the groundwater and surface water of the Lombardy region. In particular, the study focuses on the co-occurrence of different heavy metals in the same location since this aspect is particularly important in view of potential synergistic effects.

The main limitations of the study stem from the characteristics of the data collected by ARPA-Lombardy. In particular, the number of heavy metals considered is restricted, available data covers only the years 2017-2020, and groundwater and surface water stations are less present in the northern part of the region.

Possible future directions of the present work can be related to the analysis and collection of new data for locations and time-resolutions not overlapping with ARPA data. In particular, both temporal ans spatial resolutions could be improved. A further direction is represented by the analysis of pollution dynamics in surface waters near particularly polluted or industrialized areas.

Globalization has led to faster access to technology, improved communication and innovation. However, this rapid development has highly impacted the ecological cycle. Europe with the EU Green Deal aims to become the first neutral institution for climate to improve the well-being and health of citizens and of future generations. This effort has been undertaken by the USA and China becoming a global issue. In this situation, it is important to have a clear knowledge of global pollution. In particular, water is one of the most important aspect to be considered since clean water is needed for many different reasons: to avoid health problem, to have healthy soil and food and to preserve the biodiversity. The present paper proposes a method to build a picture of water contamination by heavy metals and to identify areas where action is needed. Moreover, it also helps to create a strategy for constant monitoring over time that could inform future policy decisions.

## Declarations

### Author contribution statement

Marco Zanchi, Stefano Bocchi, Stefano Gomarasca, Chiara Zanotti, Tullia Bonomi, Marco Rotiroti, Stefano Zapperi, Caterina LaPorta: Analysed and interpreted the data.

Marco Zanchi: Performed the experiments.

Caterina LaPorta: Conceived and designed the experiments; Wrote the paper.

### Funding statement

Stefano Bocchi was supported by 10.13039/501100010433Ministero dell'Ambiente e della Tutela del Territorio e del Mare [MiTE Snsvs2, code NP 1.24].

### Data availability statement

Data included in article/supp. material/referenced in article.

### Declaration of interests statement

The authors declare no conflict of interest.

### Additional information

No additional information is available for this paper.
